# High suicidality predicts overdose events among people with substance use disorder: A latent class analysis

**DOI:** 10.3389/fpubh.2023.1150062

**Published:** 2023-05-16

**Authors:** Renae D. Schmidt, Viviana E. Horigian, Dikla Shmueli-Blumberg, Kathryn Hefner, Judith Feinberg, Radhika Kondapaka, Daniel J. Feaster, Rui Duan, Sophia Gonzalez, Carly Davis, Ashley Vena, Rodrigo Marín-Navarrete, Susan Tross

**Affiliations:** ^1^Department of Public Health Sciences, University of Miami Miller School of Medicine, Miami, FL, United States; ^2^The Emmes Company, LLC, Rockville, MD, United States; ^3^Departments of Behavioral Medicine and Psychiatry and Medicine/Infectious Diseases, West Virginia University School of Medicine, Morgantown, WV, United States; ^4^Division of Research and Translational Education, Centros de Integración Juvenil A.C, Mexico City, Mexico; ^5^Department of Psychiatry, Columbia University Irving Medical Center, New York, NY, United States

**Keywords:** suicidality, substance use disorder, overdose, co-occurring disorders, substance use, suicide risk

## Abstract

**Introduction:**

Suicide is the tenth leading cause of death in the United States and continues to be a major public health concern. Suicide risk is highly prevalent among individuals with co-occurring substance use disorders (SUD) and mental health disorders, making them more prone to adverse substance use related outcomes including overdose. Identifying individuals with SUD who are suicidal, and therefore potentially most at risk of overdose, is an important step to address the synergistic epidemics of suicides and overdose fatalities in the United States. The current study assesses whether patterns of suicidality endorsement can indicate risk for substance use and overdose.

**Methods:**

Latent class analysis (LCA) was used to assess patterns of item level responses to the Concise Health Risk Tracking Self-Report (CHRT-SR), which measures thoughts and feelings associated with suicidal propensity. We used data from 2,541 participants with SUD who were enrolled across 8 randomized clinical trials in the National Drug Abuse Treatment Clinical Trials Network from 2012 to 2021. Characteristics of individuals in each class were assessed, and multivariable logistic regression was performed to examine class membership as a predictor of overdose. LCA was also used to analyze predictors of substance use days.

**Results:**

Three classes were identified and discussed: Class (1) Minimal Suicidality, with low probabilities of endorsing each CHRT-SR construct; Class (2) Moderate Suicidality, with high probabilities of endorsing pessimism, helplessness, and lack of social support, but minimal endorsement of despair or suicidal thoughts; and Class (3) High Suicidality with high probabilities of endorsing all constructs. Individuals in the High Suicidality class comprise the highest proportions of males, Black/African American individuals, and those with a psychiatric history and baseline depression, as compared with the other two classes. Regression analysis revealed that those in the High Suicidality class are more likely to overdose as compared to those in the Minimal Suicidality class (*p* = 0.04).

**Conclusion:**

Suicidality is an essential factor to consider when building strategies to screen, identify, and address individuals at risk for overdose. The integration of detailed suicide assessment and suicide risk reduction is a potential solution to help prevent suicide and overdose among people with SUD.

## 1. Introduction

Suicide is the 10th leading cause of death in the United States and is a contributor to premature mortality (1). The significance and recognition of this preventable public health problem has led to national prevention programs focused on screening, management of people at risk, and treatment (2). Suicide risk is elevated among individuals with substance use disorders (SUD) and most prevalent in patients with co-occurring substance use and mental health conditions (3). Heightened suicidal risk in this patient population makes them more susceptible to poor substance use related outcomes including intentional and unintentional overdoses. Suicidal thoughts might increase the risk of non-fatal overdose and potentially elevate the risk for future intentional overdose or unintentional overdose. Detecting individuals with SUD who are suicidal, and therefore potentially most at risk of overdose, is an essential step to address the synergistic epidemics of suicides and overdose fatalities in the United States. Because suicidal ideation and intent may underlie many overdose events (4), studies have shed light on the importance of deploying specific prevention strategies to individuals with suicidal risk and intent (5–7). Stover and colleagues investigated associations between previous overdose events and suicidal ideation, as assessed by the Self-Injurious Thoughts and Behaviors Interview and the Suicide Behaviors Questionnaire, finding that individuals with a history of suicidal intent, suicidal ideation, and overdose have greater clinical severity than those without, and recommend screening for suicidality among all overdose patients (7). In addition to those mentioned above, several tools are used to assess individuals for suicidal risk (8, 9). One validated, comprehensive screening tool for suicidality is the Concise Health Risk Tracking Self-Report (CHRT-SR), which measures thoughts and feelings associated with suicidal propensity—including constructs of helplessness, pessimism, lack of social support, and despair—and suicidal thoughts (10).

Few researchers have looked into the relationship between suicidal ideation and overdose. Cleland et al. (11) demonstrated that veterans with suicidal ideation higher than the sample average, as assessed by eight suicidal ideation items, had an additional day or more of overdose risk behaviors. In a previous study, we documented that continuous score of the CHRT-SR at baseline predicted overdose events in patients with SUD (12). The current secondary analysis was conducted across the same eight clinical trials that were conducted by the National Institute on Drug Abuse National (NIDA) Drug Abuse Treatment Clinical Trials Network (CTN) that was the focus of the prior study. We hypothesized that characterizing the levels of risk by categorizing individuals based on patterns of their item level CHRT-SR responses would allow us to further distinguish those needing intervention. Latent class analysis (LCA) is a structural equation modeling technique that facilitates identification of unobserved subgroups of individuals within a population based on responses to a set of observed variables; it assumes that individuals can be categorized by patterns of responses which relate to profiles of personal and/or environmental attributes (13). The aim of this study is to determine whether patterns of responses to the CHRT-SR can identify higher risk for overdose and other poor substance use related outcomes. It is hypothesized that individuals in the classes characterized by higher probabilities of endorsing more numerous and severe suicidal propensity items and suicidal thoughts will be more likely to overdose. It is also hypothesized that such individuals will have a higher proportion of substance use days.

## 2. Methods

The study uses data from 8 randomized clinical trials conducted by the CTN from 2012 to 2021 that used the CHRT-SR as a measure to assess suicidality of participants at baseline. The eight trials are representative of diverse interventions, targeting different drugs of choice, and recruiting from distinct settings which presents the opportunity to assess and interpret the relationship between suicidality and substance use outcomes across a broad array of patients with SUD, increasing generalizability of findings. The CTN is a network that provides an infrastructure for NIDA, medical and substance use treatment providers, academic centers, researchers, and patients to cooperatively test and deliver treatment and service options for patients with SUD (14). All data in this study were approved for public release or were publicly available on the NIDA Data Share website [https://datashare.nida.nih.gov/ (accessed on February 15, 2022)], an electronic environment that allows access to data from completed trials to promote new research using secondary analyses (15).

### 2.1. Participants

The trials included 2,543 participants (16–23); 2,541 participants were included in the current analysis (2 participants were excluded due to missing data). While each of these 8 multisite trials secured approval from their respective Institutional Review Board, the current study only used de-identified data and therefore was exempt from IRB review. Characteristics of each trial are described in Supplementary Table 1.

### 2.2. Variables

#### 2.2.1. Classification measure: Suicidality

Suicidality was assessed as the independent variable *via* baseline responses to 12 items of the CHRT-SR measured by a 5-point Likert scale ranging from Strongly disagree (1) to Strongly agree (5). To be included in the LCA, each response was coded as a binary variable; responses *Strongly Disagree, Disagree,* and *Neutral* were coded as 0 (not present) and *Strongly Agree* and *Agree* were coded as 1 (present). Items assessing suicidal propensity include prompts such as “I feel as if things are never going to get better,” “There is no one I can depend on,” and “I feel that there is no reason to live.” Items assessing suicidal thoughts include prompts such as “I have thoughts about how I might kill myself.” Cronbach’s alpha (an index of internal consistency reliability) for CHRT-SR was acceptable for all trials (CTN0037: 0.86; CTN0049: 0.91; CTN0051: 0.87; CTN0053: 0.89; CTN0054: 0.87; CTN0064: 0.90; CTN0067: 0.86; CTN0068: 0.89).

#### 2.2.2. Outcomes: Overdose and substance use days

Two outcomes assessed differences among the identified classes: substance use and overdose. An overdose event during the study period was defined as a binary outcome (Present: yes/Absent: no). Overdose included intentional and unintentional overdose. Overdose events were captured by different assessments depending on the trial. For six of the trials (CTN0037, 0051, 0053, 0054, 0067, 0068), Adverse Event forms were reviewed for MedDRA-Preferred Terms indicative of an overdose and were verified by a panel of study co-authors (VEH, RDS, DB, KH, JF, ST) through a rigorous adjudication process informed by a CTN medical monitor (RK). The key recommended term for adverse event forms was “overdose,” but related terms such as acute amphetamine toxidrome, respiratory depression and drug intoxication were considered where the term overdose was also suspected. In these cases, the medical monitor reviewed form narratives for any indication of involved substances which were then discussed by the panel to reach consensus on the adjudication of the outcome as an overdose event or not. For the other two trials (CTN 0049 (17, 24) and CTN 0064 (21, 25)), the panel similarly reviewed all causes for hospitalization and the primary cause of death, to determine whether an overdose had occurred. Key terms used to search hospitalization events (within the primary discharge diagnosis) included “Overdose,” “Abuse,” “Intoxication,” and “Detox, while deaths due to overdose were listed as “Drug Use/Overdose” or “Substance Use.” (An in-depth explanation of the process has been published (12)).

Substance use was operationalized as the proportion of assessed days wherein the participant endorsed using substances. Substance use endorsement was captured by two assessments: six trials (CTN0037, 0051, 0053, 0054, 0067, 0068) used the Timeline Follow Back [TLFB (26, 27)] which assesses daily self-reported drug and alcohol use in a specified time frame (e.g., since the last visit). The other two (CTN0049 and CTN0064) used the drug and alcohol use module of the Addiction Severity Index-Lite [ASI Lite (28)], a structured clinical interview which captures substance use over the past 30 days. While assessments were administered at different timepoints in each trial, the proportions of substance use days were calculated as the cumulative number of days endorsing any substance use divided by the total number of days for which they completed an assessment after randomization (for example, if participant X completed 5 TLFB assessments each asking about the past 7 days, then the denominator for participant X is 5 assessments*7 days = 35 total assessed days). This calculation was multiplied by 100 to create a proportion of days out of 100% per participant.

#### 2.2.3. Covariates

Several covariates were included in the models. Self-reported demographic characteristics included age, gender, and race/ethnicity. In addition, several covariates critical to the predictor and/or outcome variables were created by harmonizing several assessments across the eight trials. Each distinct assessment was operationalized into a binary indicator within its respective trial based on established thresholds or clinical significance, before being appended together with the other trials (please see Table 1 for a list of assessments included in each variable). Baseline depression was included given the correlation between depression and suicidality. A binary indicator of depression (present/absent) was created from each trial’s specific assessment including the 9-item Patient Health Questionnaire [PHQ-9 (29): CTN0067 (22, 30), CTN0068 (23, 31)], the 18-item Brief Symptom Inventory [BSI-18 (32): CTN0049 (17, 24), CTN0064 (21, 25)], the Addiction Severity Index Lite [ASI Lite (28); CTN0037 (16, 33), CTN0051 (18, 34)], the Medical and Psychiatric History [CTN0054 (20, 35)], and the Hospital Anxiety and Depression Scale [HADS (36): CTN0053 (19, 37)]. Several factors known to increase overdose risk were also included as binary variables: lifetime heroin use (38) (except for CTN0054 which did not assess this), recent alcohol and benzodiazepine use (39), and past psychiatric history (40). The assessment of lifetime use of heroin included the Addiction Severity Index Lite [ASI Lite (28): CTN0037 (16, 33), CTN0049 (17, 24), CTN0051 (18, 34), CTN0064 (21, 25), CTN0067 (22, 30)] and the Alcohol and Substance History [CTN0054 (20, 35), CTN0068 (23, 31)]. The assessment of alcohol and benzodiazepine use was determined by the ASI Lite [CTN0037 (16, 33), CTN0049 (17, 24), CTN0051 (18, 34), CTN0064 (21, 25), CTN0067 (22, 30)], and the DSM-5 checklist (41) [CTN0054 (20, 35), CTN0053 (19, 37), CTN0068 (23, 31)]. Psychiatric history excluding depression was included as a binary variable; assessments included the Medical History Form [CTN0051 (18, 34), CTN0054 (20, 35), CTN0067 (22, 30), CTN0068 (23, 31)], ASI Lite [CTN0037 (16, 33)], Additional Psychiatric Diagnosis Form [CTN0064 (21, 25)], Initial Hospital Admission Form to identify comorbid psychiatric diagnoses [CTN0049 (17, 24)], the Service Utilization Detail Form to identify individuals reporting professional help for psychological or emotional issues [CTN0049 (17, 24)], and the Mini International Neuropsychiatric Interview, version 6.0 [MINI 6.0 (42): CTN0053 (19, 37)]. Treatment arm (experimental or control) was included to account for the difference in treatment exposure within trials. Finally, each trial was included as a covariate to account for diverse study treatments, settings, targeted substance use disorders and specific populations. A detailed description of each variable’s assessment tool and operationalization has been published (12).

**Table 1:** Variable assessment forms.

Variables	Assessment forms
Predictor: Suicidality	Concise health risk tracking self-report (CHRT-SR; all 8 trials)
Outcome #1: Overdose (Y/N)	Adverse event forms (CTN0037, 0051, 0053, 0054, 0067, 0068) Hospitalization events and deaths (CTN 0049, 0064)
Outcome #2: Percentage of substance use days endorsed	Timeline follow back (TLFB; CTN0037, 0051, 0053, 0054, 0067, 0068) Addiction severity index-lite (ASI Lite; CTN0049, 0064)
Baseline depression (Y/N)	9-Item patient health questionnaire (PHQ-9; CTN0067, CTN0068) 18-Item brief symptom inventory (BSI-18; CTN0049, CTN0064) ASI Lite (CTN0037, CTN0051) Medical and psychiatric history (CTN0054) Hospital anxiety and depression scale (HADS; CTN0053)
Recent alcohol use (Y/N)	ASI lite (CTN0037, CTN0049, CTN0051, CTN0064, CTN0067) DSM-5 checklist (CTN0054, CTN0053, CTN0068)
Recent Benzo use (Y/N)	ASI lite (CTN0037, CTN0049, CTN0051, CTN0064, CTN0067) DSM-5 checklist (CTN0054, CTN0053, CTN0068)
Lifetime heroin use (Y/N)	ASI lite (CTN0037, CTN0049 CTN0051, CTN0064, CTN0067) Alcohol and substance history (CTN0054, CTN0068)
Psychiatric history (Y/N)	Medical history form (CTN0051, CTN0054, CTN0067, CTN0068) ASI lite (CTN0037) Additional psychiatric diagnosis form (CTN0064) Initial hospital admission form (CTN0049) Service utilization detail form (CTN0049) Mini international neuropsychiatric interview, version 6.0 (MINI 6.0: CTN0053).

### 2.3. Analytic plan

After each of the 12 CHRT-SR items was operationalized into binary variables as described above, the LCA was conducted to identify “classes” based on similar patterns of responses. Models with two to five classes were estimated using robust maximum likelihood (43). The models were evaluated based on several fit indices recommended by Nylund et al. (44) including the Akaike Information Criterion [AIC (45)], the Bayesian Information Criterion [BIC (46)], the sample size adjusted Bayesian Information Criterion [ssaBIC (47)], entropy, and the Lo–Mendell–Rubin Likelihood Ratio Test [LMR-LRT (48)]. Additionally, estimated probabilities, plot/plot interpretability, and sample size of each class (49) were considered in selection of the final model.

Descriptive statistics, including mean and standard deviation for continuous variables and frequencies and proportions for categorical variables, were calculated for participants overall and by class. A multivariate logistic regression, using a generalized estimating equation, analyzed class membership as a predictor of overdose, while controlling for covariates. A beta-binomial finite mixture model analyzed class membership as a predictor of substance use days, controlling for covariates. A beta-binomial was used to account for the bi-modal nature of substance use days (50). Adjusted odds ratios and 95% confidence intervals were calculated. While LCA addresses missing data *via* maximum likelihood estimates, missing data were excluded from final analyses as the generalized estimating equations ignore any observation with a missing value for any variable. For all analyses, two-tailed value of ps less than 0.05 were considered statistically significant. The LCA was conducted using Mplus 6.1 (51). All other analyses were performed using SAS version 9.4 (52).

## 3. Results

A total of 2,541 participants were included in this analysis. Characteristics included mean age of 39.4 (SD 11.4) years, 67.4% were male sex, 41.3% were White individuals, 38.3% were Black individuals, and 14.4% were Hispanic individuals. Approximately half of the sample (50.2%) indicated that they had at least one preexisting psychiatric diagnosis, and 51.6% scored in the depressed range at baseline. With regards to substance use, 60.0% reported recent use of alcohol, 15.8% reported recent use of benzodiazepines, and 39.0% reported lifetime use of heroin. Seventy-five participants (3.0%) had at least one overdose event during their study participation (Table 2). Demographic information by study can be found in the primary publications for each one (16–23). The total number of participants varied slightly due to occasional missing data.

**Table 2:** Participant characteristics overall and by class.

		Overall		Class 1: Minimal suicidality		Class 2: Moderate suicidality		Class 3: Highest suicidality		Value of *p*
Total		2,541		1884		471		186		-
Age in years		39.0	11.0	39.0	11.6	39.7	11.0	42.9	10.3	<0.0001
Gender	Female	829	32.6%	592	31.4%	187	39.7%	50	26.9%	0.0006
	Male	1712	67.4%	1,292	68.6%	284	60.3%	136	73.1%	
Race/ethnicity	Black/Afr Am	972	38.3%	742	39.4%	143	30.4%	87	46.8%	0.0022
	Hispanic	366	14.4%	269	14.3%	71	15.1%	26	14.0%	
	Other	153	6.0%	114	6.1%	31	6.6%	8	4.3%	
	White	1,050	41.3%	759	40.3%	226	48.0%	65	34.9%	
Treatment arm	Control	1,231	48.4%	917	48.7%	233	49.5%	81	43.5%	0.3937
	Experimental	1,310	51.6%	967	51.3%	238	50.5%	105	56.5%	
Psychiatric history	No	1,265	49.8%	1,012	53.7%	182	38.6%	71	38.2%	<0.0001
	Yes	1,276	50.2%	872	46.3%	289	61.4%	115	61.8%	
Lifetime heroin use	Missing	304	12.0%	267	14.2%	32	6.8%	5	2.7%	<0.0001
	No	1,245	49.0%	921	48.9%	210	44.6%	114	61.3%	
	Yes	992	39.0%	696	36.9%	229	48.6%	67	36.0%	
Recent alcohol use	No	1,016	40.0%	742	39.4%	195	41.4%	79	42.5%	0.5235
	Yes	1,523	59.9%	1,142	60.6%	275	58.4%	106	57.0%	
Recent Benzo use	No	2,139	84.2%	1,613	85.6%	371	78.8%	155	83.3%	0.0018
	Yes	400	15.7%	271	14.4%	99	21.0%	30	16.1%	
Overdose	No	2,453	96.6%	1825	96.9%	449	95.3%	179	96.2%	0.2286
	Yes	87	3.4%	58	3.1%	22	4.7%	7	3.8%	
Depressed at baseline	Yes	1,310	51.6%	806	42.8%	345	73.2%	159	85.5%	<0.0001
	No	1,230	48.4%	1,078	57.2%	125	26.5%	27	14.5%	
Substance use days (% out of 100)		44.8	38.1	42.0	37.9	54.2	37.0	49.1	38.0	<0.0001

*N* and % are shown for categorical variables, mean and standard deviation are shown for continuous variables.

Latent class analysis model fit was assessed for models with 2–5 classes (Table 3). Multiple fit statistics and interpretability indicated that a 3-class model best fit the data. Both the BIC and the sample-size adjusted BIC scores were lower in the 3-class model than the 2-class model, while maintaining a higher entropy than the 4-class model. The 3-class model also presented a solution with a logical substantive interpretation, with adequate class distinction and sample sizes. The 5-class model had classes with fewer than 5% of the sample.

**Table 3:** Latent class analysis model fit statistics.

Model	Log likelihood	AIC	BIC	ssaBIC	LMR-LRT	LMR-LRT value of *p*	Entropy
2-class	−9273.771	18597.543	18743.550	18664.119	3927.882	<0.0001	0.892
**3-class**	**−9044.868**	**18165.736**	**18387.668**	**18266.932**	**453.358**	**<0.0001**	**0.874**
4-class	−8929.231	17960.462	18258.318	18096.278	229.027	0.009	0.822
5-class	−8864.552	17857.104	18230.884	18027.539	128.102	0.003	0.837

AIC, Akaike Information Criterion; BIC, Bayesian Information Criterion; LMR-LRT, Lo–Mendell–Rubin likelihood ratio test; ssaBIC, sample size adjusted Bayesian Information Criterion.The bold values represent the final selected model.

**Figure 1:**
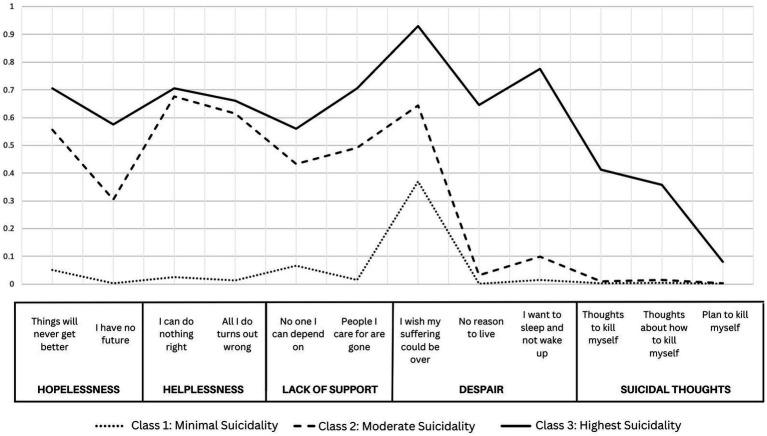
Probabilities of class membership for a three-class solution of item-level responses to CHRT-SR.

The selected model presents three unique groups of individuals based on baseline responses to the CHRT-SR. These were labeled Class (1) *Minimal Suicidality*, with low probabilities of endorsing each of the constructs; Class (2) *Moderate Suicidality*, with high probabilities of endorsing pessimism, helplessness, and lack of social support, but minimal endorsement of despair or suicidal thoughts; and Class (3) *Highest Suicidality* with high probabilities of endorsing all constructs. Figure 1 shows the probabilities of endorsing each item by most-likely class membership in this 3-class model.

Class 1, the *Minimal Suicidality* class, comprised of 1,884 participants—or 74.1% of the overall sample—was the largest of the three classes. Individuals in this class were 68.6% male, 39.4% identified as Black/African American, and 41.3% identified as White. Compared with the other two classes, this class comprised the lowest proportions of those with psychiatric history (46.3%), baseline depression (42.8%), and recent use of benzodiazepines (14.4%). It also had the lowest proportion of individuals with an overdose event (2.8%) and the lowest average endorsement of substance use days at 42.0% of assessed days during trial participation. However, they had the highest proportion of individuals with recent alcohol use (60.6%).

Class 2, the *Moderate Suicidality* class, was the second largest class comprised of 471 participants (18.5%). Relative to the other two classes, this class had the highest proportion of females (39.7%), White (48.0%) and Hispanic individuals (15.1%), and those with lifetime heroin use (48.6%). It had the second highest proportion of individuals with baseline depression (73.2%), psychiatric history (61.4%), and overdose (3.4%). Individuals in this class had the highest average endorsement of substance use days at 54.2% of assessed days.

Class 3, *Highest Suicidality* class, had the smallest class membership, with 186 participants (7.3%). Relative to the other two classes, the Highest Suicidality class had the highest proportions of males (73.1%) and Black/African American individuals (46.8%) and the highest rates of psychiatric history (61.8%) and baseline depression (85.5%). They also had the highest proportion of individuals with an overdose event (3.8%). They endorsed substance use during an average 49.1% of assessed days.

Results of the first logistic regression analysis (shown in Table 4) reveal that class membership was associated with overdose events as those in the Highest Suicidality class were more likely to overdose as compared to those in the Minimal Suicidality class (OR = 1.45; 95% CI = 1.02, 2.05). Lifetime heroin use was also associated with increased odds of overdose (OR = 2.76; 95% CI = 1.85, 4.12). Black/African American individuals (OR = 0.64, 95% CI = 0.42,0.97) and Other race/ethnicity (OR = 0.33, 95% CI = 0.19,0.56) as compared to White individuals, and those with recent alcohol use (OR = 0.81, 95% CI = 0.69,0.96) were less likely to overdose.

**Table 4:** Results of logistic regression/generalized estimating equation assessing class membership as a predictor of overdose.

		Reference	Odds ratio (95% CI)	Value of *p*
Class	Moderate suicidality (2)	Minimal suicidality (1)	1.11 (0.82–1.50)	0.51
	Highest Suicidality (3)		1.45 (1.02–2.05)	0.04
Age			1.00 (0.98–1.02)	0.77
Gender	Female	Male	0.74 (0.36–1.50)	0.40
Race/ Ethnicity	Black/Afr Am	White	0.64 (0.42–0.97)	0.03
	Hispanic		1.23 (0.69–2.21)	0.48
	Other		0.33 (0.19–0.56)	<0.0001
Depressed	Yes	No	0.81 (0.33–1.97)	0.64
Recent Alcohol Use	Yes	No	0.81 (0.69–0.96)	0.02
Recent Benzo Use	Yes	No	1.41 (0.88–2.27)	0.15
Lifetime Heroin Use	Missing	No	0.16 (0.10–0.25)	<0.0001
	Yes		2.76 (1.85–4.12)	<0.0001
Psychiatric History	Yes	No	0.80 (0.60–1.05)	0.11
Treatment Arm	Experimental	Control	1.30 (0.80–2.12)	0.29

Results of the beta-binomial finite mixture model analysis (Table 5) reveal that class membership was not associated with substance use days. However, recent alcohol use and lifetime heroin use were associated. Those who endorsed recently using alcohol (OR = 1.25; 95% CI = 1.01,1.53) and those who endorsed using heroin in their lifetime (OR = 1.99; 95% CI = 1.51,2.63) were more likely to have a higher proportion of substance use days as compared to those who did not endorse using these substances.

**Table 5:** Results of beta-binomial finite mixture model assessing class membership as a predictor of substance use days.

		Reference	Odds ratio (95% CI)	Value of *p*
Class	Moderate suicidality (2)	Minimal suicidality (1)	1.10 (0.85–1.42)	0.45
	Highest suicidality (3)		1.00 (0.69–1.46)	0.99
Age			0.99 (0.99–0.99)	
Gender	Female	Male	0.97 (0.79–1.20)	0.80
Race/ethnicity	Black/Afr Am	White	0.96 (0.73–1.26)	0.78
	Hispanic		1.03 (0.76–1.38)	0.86
	Other		1.03 (0.68–1.56)	0.89
Depressed	Yes	No	1.09 (0.87–1.36)	0.45
Recent alcohol use	Yes	No	1.25 (1.01–1.53)	0.04
Recent Benzo use	Yes	No	1.06 (0.79–1.41)	0.71
Lifetime heroin use	Yes	No	1.99 (1.51–2.63)	<0.0001
Psychiatric history	Yes	No	0.91 (0.71–1.17)	0.46
Treatment arm	Experimental	Control	1.10 (0.90–1.34)	0.36

## 4. Discussion

Results of this study demonstrate that class type, based on responses to the 12-item CHRT-SR that characterizes suicidality, was associated with overdose. Individuals in the Highest Suicidality class, who were categorized by their high probabilities of endorsing all suicidality domains, were more likely to overdose than those in the Minimal Suicidality class. As opposed to analysis of the continuous CHRT-SR score, the LCA depicts the domains of suicidality that were endorsed by each category of individuals. For example, the Highest Suicidality class was the only class to specifically endorse suicidal thoughts in addition to each of the items assessing suicidal propensity (helplessness, lack of social support). Previous work by Gicquelais et al. demonstrated survivors of opioid overdose events had suicidal intent and feelings of apathy toward risk of overdose. The results of this study indicated that individuals who recalled suicidal intent linked to their overdose event were at increased risk of suicide or self-harm during SUD treatment (5). However, to our knowledge the current study is the first study to evaluate patterns of responses to the CHRT-SR to predict overdose events, therefore limiting possible comparison with existing literature. Of concern, among the three classes, the Highest Suicidality class had the largest representation of Black/African American individuals, highlighting the disparities typically experienced by this group. Both Ivey-Stephenson et al. as well as Joe et al. have also demonstrated higher levels of suicidal thoughts and attempts in Black/African American individuals as compared to other demographic groups (53, 54). While the current study was not aimed at uncovering the causes of these responses, it is possible that these findings are, at least in part, reflective of the social determinants of health and structural health disparities. For example, racial discrimination (55, 56) and inadequate access to healthcare (57, 58) which are more prevalent among Black/African American individuals, are also associated with suicidal ideation.

Notably, and consistent with the literature, results also showed that lifetime heroin use was strongly associated with overdose. Others have demonstrated an increased risk of opioid overdose events among individuals with a history of heroin (7, 59). As may be expected, alcohol use and heroin use were associated with a higher proportion of substance use days, though finite mixture modeling did not demonstrate a significant relationship between Suicidality class and substance use days after controlling for variability by protocol. Nonetheless, suicidality may be an important factor impacting overall substance use and warrants further investigation.

Validated thresholds of the CHRT-SR have not been established; the instrument’s existing interpretation is based on continuous scores. In the current analysis, we examined stratified mean CHRT-SR scores by latent class: the mean score among those in the Minimal Suicidality class was 20.15 (SD 5.51; min 11-max 36), among those in the Moderate Suicidality class was 31.85 (SD 4.83; min 18-max 48), and finally among those in the Highest Suicidality class was 41.16 (SD 5.34; min 29-max 59). While this work did not set out to establish clinical thresholds of the CHRT-SR, this may be a direction of future work given the distinct average scores among three unique groups and the subsequent utility to identify those at higher risk for overdose. It is important to note that there are only minimal overdose events across the three classes in this analysis, however the opportunity to predict and prevent this life-threatening outcome is still meaningful. Due to the very low rate of this outcome, single trial analyses are often underpowered to consider overdose as an outcome. Validating clinical thresholds of the CHRT-SR which indicate mild, moderate, and severe risk for adverse events, including both suicide and overdose, could offer clinicians an enhanced approach to determine and address risk during screening. Particular attention should be given in cases where items of suicidal ideation are endorsed.

This examination has several strengths. First, in contrast to previous studies, we used a rigorous psychometric method, LCA, which identified meaningful profiles or types of respondents, therefore unveiling those that might be at higher risk. This is significant as it can potentially allow the development and implementation of targeted interventions tailored to these different subgroups. Secondly, this study used data across eight trials with multiple sites located in different regions of the United States and drew from a broad range of participants with SUD. The use of large datasets grants practical examinations such as this one. It also allows for greater generalizability of results to different geographical locations and participant characteristics. While these findings are post-hoc, they provide notable evidence and rationale for prospective, *a-priori* stated, hypothesis driven work. This study also presents several limitations. First, its design only allowed for examination of associations and not causation. Second, the population assessed in this examination is representative of individuals seeking treatment for SUD who agreed to participate in a research study. Therefore, our results might not be generalizable to the entire population of persons with SUD. Third, while a process of consensus was used to ascertain the outcome of overdose and all other covariates, there was notable heterogeneity in instruments used across trials. Fourth, a limitation of the LCA includes the potential loss of information recoding continuous indicators of the CHRT-SR into categorical variables. Finally, the operationalization of drug use days relied on the number of assessed days, which differed across trials. This approach also is limited by the lack of information on unassessed days. Some of these limitations are the tradeoffs when harmonizing large data sets.

While this study did not consider polysubstance use, evidence suggests that concurrent use of multiple substances is common among people with SUD (60). For example, among individuals in treatment for opioid use disorder (OUD), rates of polysubstance use range between 65% (61) to 85% (62). Research has demonstrated associations between co-use of substances and a higher risk of overdose among participants experiencing psychological distress (63), as well as an increase in reporting thoughts of self-harm during addiction treatment (5). In an investigation of 2,637 individuals enrolled across three Clinical Trials Network trials (CTN0027, 0030, 0051), Pan and colleagues saw high rates of polysubstance use and concluded that distinct patterns of polysubstance use differentially predict relapse outcomes (64). Future examination of these patterns and their relationship with overdose and other outcomes across these eight trials is warranted.

Suicide is the 4^th^ leading cause of death among individuals 35–44 years old, the population mostly represented in these substance use treatment trials (65). Suicidality is a critical factor to consider when developing strategies to screen, identify, and address individuals at risk for overdose, and could be critical in guiding an individual’s substance use treatment. The integration of detailed suicide assessment and suicide risk reduction is a key step to prevent poor outcomes among people with SUD. A holistic approach to addressing mental health conditions at the person-level is critical. On a public health level, only a concerted approach will help address the current synergistic epidemics of suicidality and overdose deaths in the US.

## Data availability statement

TN trial data is publicly available on NIDA Data Share: https://datashare.nida.nih.gov/. Data for the trials included in this study are available as follows: CTN0037 https://datashare.nida.nih.gov/study/nida-ctn-0037, CTN0049 https://datashare.nida.nih.gov/study/nidactn0049, CTN0051 https://datashare.nida.nih.gov/study/nidactn0051, CTN0053 https://datashare.nida.nih.gov/study/nidactn0053, CTN0054 https://datashare.nida.nih.gov/study/nidactn0054, CTN0068 https://datashare.nida.nih.gov/study/nidactn0068. Investigators interested in obtaining data for CTN0064 and CTN0067 may direct inquiries to the corresponding author.

## Ethics statement

The studies involving human participants were reviewed and approved by the respective Institutional Review Board for each of the 8 multisite trials (however the current study only used de-identified data and therefore was exempt from ethical review). All participants provided their written consent to participate in their respective trial.

## Author contributions

VH formulated the research question and drafted the first version of the manuscript. DS-B, KH, JF, ST, VH, and RS reviewed and decided on measures for all covariates across trials. DF provided methodological guidance and approach for the model created for data analysis. RS drafted the methods section and performed the analyses, with support of RD. DS-B, KH, JF, ST, RK, VH, and RS are the expert panel members who adjudicated the outcomes in the initial manuscript. SG and CD conducted literature searches and background review, and contributed to the referencing system. All authors contributed to the article and approved the submitted version.

## Funding

Research reported this publication was supported by the National Institute on Drug Abuse of the National Institutes of Health under award numbers UG1DA013720, 2UG1DA049436, U54GM104942, UG1DA013035, 2UG1DA049436-01,75N95019D00013/N01DA-19-2250 (NIDA DSC contract to The Emmes Company, LLC), and 75N95020D00012/N01DA-20-2251 (NIDA CCC contract to The Emmes Company, LLC). The content is solely the responsibility of the authors and does not necessarily represent the official views of the National Institutes of Health.

## Conflict of interest

DS-B, KH, RK, and AV were employed by the company The Emmes Company, LLC.

The remaining authors declare that the research was conducted in the absence of any commercial or financial relationships that could be construed as a potential conflict of interest.

## Publisher’s note

All claims expressed in this article are solely those of the authors and do not necessarily represent those of their affiliated organizations, or those of the publisher, the editors and the reviewers. Any product that may be evaluated in this article, or claim that may be made by its manufacturer, is not guaranteed or endorsed by the publisher.
