# Supplementary material for: High suicidality predicts overdose events among people with substance use disorder: A latent class analysis
Source: Front Public Health. 2023 May 16;11:1150062. doi: 10.3389/fpubh.2023.1150062 (PMC10228506; doi:10.3389/fpubh.2023.1150062)
Supplement: Supplementary file 1 [file Table_1.DOCX]

Supplementary Table 1. Selected CTN Trial Characteristics.

| **Trial** | **Study Title** | **Study Type** | **Sample Size** | **Main Target Substance** | **Recruitment Setting** | **Intervention period/ Follow Up period** |
| --- | --- | --- | --- | --- | --- | --- |
| CTN 0037(15, 32) | Stimulant Reduction Intervention Using Dosed Exercise (STRIDE) | 2-arm RCT | 302 | Stimulants (Cocaine and Methamphetamine) | Residential substance use treatment programs | 12 weeks/  36 weeks |
| CTN 0049(16, 23) | Project HOPE: Hospital Visit as Opportunity for Prevention and Engagement for HIV-Infected Drug Users | 3-arm RCT | 801* | Any substance | Inpatient, Hospitalized, enrolled at bedside | 26 weeks/  52 weeks |
| CTN 0051(17, 33) | Extended-Release Naltrexone vs Buprenorphine for Opioid Treatment (X:BOT) | 2-arm comparative effectiveness RCT | 570 | Opioids | Community based treatment programs | 24 weeks /  36 weeks |
| CTN 0053(18, 36) | Achieving Cannabis Cessation: Evaluating N-Acetylcysteine Treatment (ACCENT) | Double-blind, placebo controlled 2-arm RCT | 302 | Cannabis | Multicenter, "treatment-seeking cannabis-dependent adults who submit positive urine cannabinoid testing during screening" | 12 weeks/  16 weeks |
| CTN 0054(19, 34) | Accelerated Development of Additive Pharmacotherapy Treatment (ADAPT) | 2-stage pilot study | 49 | Methamphetamine | Outpatient, community treatment programs | 8 weeks/  9 weeks |
| CTN 0064(20, 24) | **Linkage to Hepatitis C Virus (HCV) Care among HIV/HCV Co-infected Substance Users** | 2- arm RCT | 113 | Any substance | Follow up population of CTN 0049 | 26 weeks/  52 weeks |
| CTN 0067(21, 29) | Comparing Treatments for HIV-Infected Opioid Users in an Integrated Care Effectiveness Study (CHOICES) Scale-Up | 2-arm RCT | 116 | Opioids | Primary Care | 24 weeks/  24 weeks |
| CTN 0068(22, 30) | Accelerated Development of Additive Pharmacotherapy Treatment (ADAPT-2) for Methamphetamine Use Disorder | Double-blind, placebo controlled 2-arm RCT with adaptive design | 403 | Methamphetamine | Adults 18-65 were recruited from communities near the trial sites with the use of ads and direct referrals | 12 weeks/  16 weeks |

RCT: Randomized Controlled Trial. *The CTN 0049 population assessed in this analysis includes only those unduplicated patients (N=688 of 801 total) who were not re-randomized to CTN 0064 (N=113).
